# Serum Uric Acid Is Associated with Metabolic Syndrome and Insulin Resistance among Health Personnel from Peru

**DOI:** 10.1155/2021/9933319

**Published:** 2021-11-15

**Authors:** Brenda M. Galindo-Yllu, Ricardo Rojas-Humpire, Carlos J. Toro-Huamanchumo, Rosmery Gutierrez-Ajalcriña, Anderson N. Soriano

**Affiliations:** ^1^Clinical and Epidemiological Research Unit, School of Medicine, Universidad Peruana Union, Lima, Peru; ^2^Universidad San Ignacio de Loyola, Unidad para la Generación y Síntesis de Evidencias en Salud, Lima, Peru; ^3^Unidad de Epidemiología y Salud Ambiental, Hospital de Huaycán, Ate, Peru; ^4^Research Coordination, Clínica Good Hope, Lima, Peru

## Abstract

We explored the association between serum uric acid (SUA) and metabolic syndrome (MetS) and insulin resistance (IR) among health personnel from a public hospital in Peru in a cross-sectional study with data from the Plan for the Prevention and Surveillance of Communicable and Noncommunicable Diseases of Huaycán Hospital. MetS was defined according to Latin American Diabetes Association (ALAD) criteria and IR with surrogate IR markers, triglyceride-to-HDL-C ratio (TG/HDL-C), and triglyceride-to-glucose index (TyG). The association between SUA and MetS and IR was determined using Poisson regression models in a sample of 292 participants with an average age of 46.2 ± 10.6 years. The total prevalence of MetS was 38%, and the individuals with MetS presented mainly alterations in anthropometric parameters (obesity and body fat). Finally, the adjusted regression models showed that women with SUA in the highest tertile increased the prevalence of MetS (PR: 1.71, 95% CI: 1.07–2.74) compared to the lowest tertile of SUA in women, while SUA increased hypertriglyceridemia and IR (TG/HDL-C and TyG) in both sexes. We concluded that SUA is strongly associated with MetS in women, and SUA increases hypertriglyceridemia and IR in both sexes. On the contrary, more research is required regarding the female population.

## 1. Introduction

In recent decades, metabolic syndrome (MetS) has increased dramatically and is considered one of the most important risk factors for cardiovascular disease [1]. MetS is a set of interrelated clinical disorders, including dyslipidemia, central obesity, glucose intolerance, and high blood pressure [2]. Its presence is involved in the development of various diseases such as fatty liver, diabetes mellitus (DM), cancer, and cardiovascular and infectious diseases [3, 4]. Prior research indicates that insulin resistance (IR) plays an important role in the pathophysiology of this condition [5]. Considering this, TyG and TG/HDL ratio are simple and practical surrogate markers for insulin resistance that can be used in primary healthcare [6].

Serum uric acid (SUA) is an excretory metabolite produced by the metabolism of the purines [7]. It can be elevated as a result of the low renal filtration rate, overproduction of purine precursors, and diet [8]. SUA regulates proinflammatory pathways in vascular smooth muscle cells and oxidative stress at the mitochondrial level [9]. Additionally, it is involved in the mechanisms of metabolic dysregulation mediated by excess fructose [10]. Previous studies have reported the association of SUA concentrations and MetS, IR, DM, and other cardiometabolic diseases [11, 12].

Several Latin American countries have exhibited a high prevalence of MetS [13]. The biological diversity of the Latino population leads to changes in the prevalence and development of certain diseases such as metabolic diseases [14]. Several risk factors have been studied; for example, a study in Brazilian women found that anthropometric parameters and estradiol were strongly associated to MetS [15]; however, SUA was not evaluated in that study and could be an important factor to consider. On the contrary, healthcare personnel are important agents of public health and quality of life in communities [16]. Moreover, the work pace of health personnel, bad sleep quality, stress, and anxiety could increase the risk of cardiometabolic disorders such as diabetes, hypertension, IR, and MetS [17]. For this reason, we found it appropriate to conduct a study in a Latin population, specifically in a Peruvian hospital, to analyze the association of MetS with SUA and IR stratified by sex in health personnel.

## 2. Methods

### 2.1. Design of the Primary Study

We conducted a cross-sectional analytical study using data from adult workers of both sexes who were part of the Plan for the Prevention and Surveillance of Communicable and Noncommunicable Diseases at Hospital de Huaycán II-1, Lima, Peru, in 2019. In this prevention plan, clinical evaluation and laboratory tests are performed to prevent and diagnose diseases in workers. Trained personnel collected data on demographic characteristics, lifestyle behaviors, and anthropometric measurements and laboratory data using questionnaires. Likewise, workers were explained that their medical data would be used for future research, and written informed consent was obtained from all participants.

### 2.2. Eligibility Criteria

We included health personnel data from Hospital de Huaycan. Pregnant women, participants who did not fill out the form, participants who did not perform laboratory tests, and those who did not present plausible data for the study were excluded. We eliminated 75 observations due to missing data (*n* = 46, 12.5%) and implausible data (*n* = 29, 7.9%) (Table S1 in Supplementary Materials).

### 2.3. Definition of Variables

#### 2.3.1. Exposure: SUA

SUA concentration was categorized into tertiles according to sex-specific distribution: T1 (2.5–4.4 mg/dL), T2 (4.5–5.0 mg/dL), and T3 (5.1–6.3 mg/dL) for men and T1 (1.2–3.1 mg/dL), T2 (3.2–3.8 mg/dL), and T3 (3.9–6.8 mg/dL) for women.

#### 2.3.2. Outcome: MetS

MetS was defined according to the criteria of Latin American Diabetes Association (ALAD) 2010 [18], including waist circumference (WC), triglycerides (TG), high-density lipoprotein cholesterol (HDL-C), systolic blood pressure (SBP), diastolic blood pressure (DBP), fasting glucose (FG), glycosylated hemoglobin (HbA1c), and some data from the workers' registry. The criteria for SM were central obesity (WC ≥ 94 cm in men and ≥88 cm in women) and two or more of the following: hypertriglyceridemia (TG > 150 mg/dL or in specific hypolipidemic therapy), low HDL-C (HDL-C < 40 mg/dL in men and <50 mg/dL in women), high blood pressure (SBP ≥130 mmHg and/or SBP ≥85 mmHg or on antihypertensive treatment), or impaired glucose regulation (SBP ≥100 mg/dL, HbA1c > 5.6%, or in treatment for DM).

#### 2.3.3. Other Variables

Triglyceride-to-HDL cholesterol ratio (TG/HDL-C) and triglyceride-to-glucose index (TyG) were selected as insulin resistance indexes because they are accurate indicators of insulin resistance diagnosis in many populations [19]. TG/HDL-C was calculated using the following formula: fasting TG (mg/dL)/fasting HDL cholesterol (mg/dL), and it was categorized into elevated (≥3) and normal TG/HDL-C [20]. TyG was calculated using the formula Ln[fasting TG (mg/dL) × fasting plasma glucose (mg/dL)/2] and categorized into elevated (≥8.65) and normal TyG [21].

Nutrition, smoking, alcohol consumption, and physical activity were extracted from the Fantastico, a healthy lifestyle questionnaire, validated in Peru [22]. The variables were categorized into “good” nutrition (balanced diet almost always and no consumption of sugar, salt, junk food, or high fat) and “bad” nutrition (balanced diet sometimes or rarely and consumption of sugar, salt, junk food, and/or high fat), “physically active” (active exercise at least 20 min four or more times per week) and “physically inactive” (active exercise at least 20 min one to three times per week or less than one time per week), “low-moderate alcohol consumption” (0–7 drinks per week) and “high alcohol consumption” (8–12 or more drinks per week), and “nonsmoking” (no smoking in the last year) and “smoking” (smoked this year or smokes 1–10 cigarettes per day or more than 10 per day). The family history of DM was extracted from the FINDRISC questionnaire and was categorized as “Yes” and “No.” The variables of age, sex, type of employee, BMI, low-density lipoprotein cholesterol (LDL-C), very low-density lipoprotein cholesterol (VLDL-C), total cholesterol, and body fat percentage were also included.

### 2.4. Statistical Analysis

Data analysis was performed with RStudio v1.3 software. Categorical variables were described in absolute and relative frequencies. Numerical variables were described with mean and standard deviation. To assess the association between the SUA level (as a numerical value and categorized by tertiles) and metabolic syndrome, prevalence ratios (PRs) and their respective 95% confidence intervals (95% CIs) were determined using Poisson regression models with robust variance. The first model examined the bivariate association between SUA and metabolic syndrome. The second model was adjusted for age and sex in the overall population and by age in the sex-stratified population. The third model was additionally adjusted for BMI variables, nutrition, smoking, alcohol consumption, physical activity, and family history of DM. In the same way, the analysis of MetS components and insulin resistance markers had adjustments for these potential confounders. The association analysis was stratified by sex, and a *p* value <0.05 was considered statistically significant.

### 2.5. Ethical Considerations

The study was approved by the Institutional Review Board of Hospital de Huaycán (no. 023-2020) and of the Faculty of Health Sciences of the Universidad Peruana Unión (no. 00136-2020).

## 3. Results

### 3.1. General Characteristics of the Study Population

We analyzed the data from a total of 292 individuals (202 women and 90 men). The mean age of the participants was 46.2 ± 10.6 years, and workers were in administrative (25.3%), patient contact (66.5%), and general services (8.2%) positions. The largest proportion was nonsmokers (87.7%), had good nutrition (71.6%), had no family history of DM (65.1%), reported low-moderate alcohol consumption (93.5%), and were physically active (81.5%). The mean concentration of SUA was 3.9 ± 1.0 mg/dL in the general population, 3.49 ± 0.88 mg/dL in women, and 4.70 ± 0.85 mg/dL in men. The characteristics of both MetS and no-MetS groups are presented in Table S2 in Supplementary Materials.

The patient contact personnel proportion was the highest in the group with MetS, with ages between 50 and 60 years. The MetS group showed important anthropometric alterations, obesity, and visceral fat. Furthermore, glucose metabolism (HbA1c: 6.6% in men and 6.2% in women; both *p* < 0.05), lipid profile, triglycerides (234 mg/dL in men and 188 mg/dL in women; both *p* < 0.05), total cholesterol (216 mg/dL in men and 199 mg/dL in women; both *p* < 0.05), VLDL-C (40.5 mg/dL in men and 36.6 mg/dL in women; both *p* < 0.05), and blood pressure presented significant differences in the MetS group compared to the no-MetS group in both sexes. However, some laboratory tests in the MetS group presented significant changes only in women, such as HDL-C (53 vs. 47.8 mg/dL, *p* < 0.01) and uric acid (3.3 vs. 3.8 mg/dL, *p* < 0.01), while only in men, changes in LDL-C (113 vs. 129 mg/dL, *p* < 0.05) were shown (Table S2 in Supplementary Materials).

### 3.2. MetS Prevalence by Tertiles of SUA Concentration

**Figure 1:**
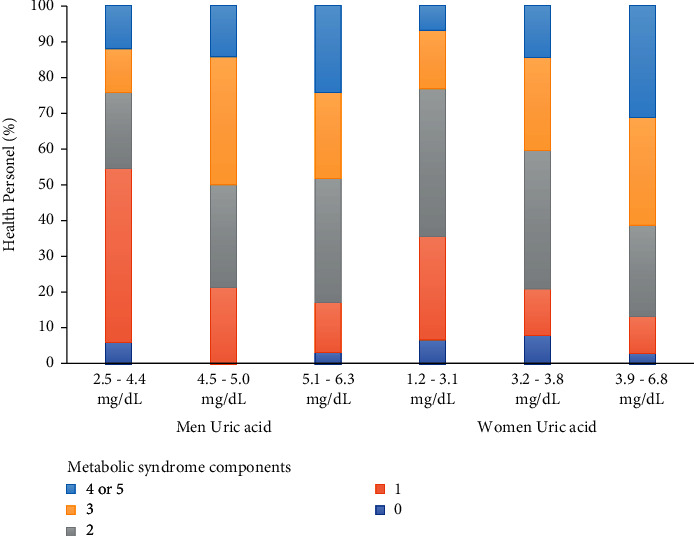
Components of metabolic syndrome frequency according to tertiles of uric acid. T1 (2.5–4.4 mg/dL), T2 (4.5–5.0 mg/dL), and T3 (5.1–6.3 mg/dL) in men; T1 (1.2–3.1 mg/dL), T2 (3.2–3.8 mg/dL), and T3 (3.9–6.8 mg/dL) in women.

The prevalence of MetS in the total population was 38%, 36.7% in men, and 38.6% in women. The components of MetS changed by each SUA tertile. In this way, more MetS components were pooled in the high tertile than in the low tertile in both men (24.1 vs. 12.1%) and women (31.3 vs. 6.8%), as shown in Figure 1.

### 3.3. Poisson Regression Models with Robust Variance to Assess the Association between SUA Tertiles and Metabolic Syndrome

All regression models are presented in Table 1. In the crude Poisson regression model to calculate the association between SUA tertiles and MetS in the overall population, compared with the low tertile group, the prevalence of MetS in the intermediate and high tertile was higher (PR = 1.64, 95% CI: 1.10–2.46; PR = 1.76, 95% CI: 1.19–2.62, respectively). Both were statistically significant. In the second model, the association maintained the direction and statistical significance (PR = 1.60, 95% CI: 1.07–2.39; PR = 2.04, 95% CI: 1.33–3.12, respectively). In the third model, there was no statistical difference between the intermediate and high tertile compared to the low tertile (PR = 1.34, 95% CI: 0.92–1.95; PR = 1.40, 95% CI: 0.96–2.05, respectively).

When stratified by sex in the second model, men had a higher prevalence of MetS for the intermediate tertile compared to the low tertile (PR = 2.36, 95% CI: 1.12–4.95), which was statistically significant. In the third model, the association maintained the pattern (PR = 1.79, 95% CI: 0.98–3.23), although there was no statistical difference. Women in the second model had a higher prevalence of MetS for the high tertile compared to the low tertile (PR = 2.43, 95% CI: 1.50–3.93), which was statistically significant. In the third model, the association maintained the pattern and statistical significance (PR = 1.71, 95% CI: 1.07–2.74).

The fully adjusted PRs for each 1 mg/dl increment in SUA concentration for MetS were 1.22 (95% CI: 1.04–1.44) in the overall population and 1.24 (95% CI: 1.01–1.52) in women. In men, no association was found.

### 3.4. Association between SUA Tertiles and MetS Components and Insulin Resistance Markers

In the adjusted Poisson regression analysis shown in Table 2, the highest tertile of SUA was significantly associated with hypertriglyceridemia (PR: 2.02, 95% CI: 1.13–3.62) in women, while in men, the middle tertile (PR: 2.27, 95% CI: 1.22–4.25) and the highest tertile of SUA (PR: 1.94, 95% CI: 1.01–3.73) presented a significant association to hypertriglyceridemia. In the same way, insulin resistance markers, TG/HDL-C and TyG index, showed elevated levels in the middle tertile of SUA in men. However, in women, only the highest tertile of SUA was associated to elevated TyG index (PR: 1.90, 95% CI: 1.30–2.76).

## 4. Discussion

In the present study, we evaluated the association of SUA to MetS and insulin resistance in health personnel from Peru. Our results showed that health personnel with MetS presented important alterations in anthropometric variables and laboratory tests. Furthermore, hypertriglyceridemia and IR were associated to SUA in both sexes after adjusted analysis. However, only women presented a significant association of SUA to MetS.

SUA is an antioxidant metabolite that maintains the stability of the vascular endothelium [7]. High SUA levels produce a pro-oxidant environment, endothelial dysfunction, and mitochondrial damage. Additionally, the increase of reactive oxygen species (ROS) and inflammatory proteins (interleukin-1, interleukin-6, and TNF-*α*) are involved in the development of IR and MetS [9]. Previous studies have shown that elevated SUA levels predispose to IR and MetS [23, 24]. Some studies infer that high SUA levels may be both a risk factor and an outcome of some metabolic disorders [25, 26].

The association of SUA to MetS was significant in women but not in men in this study. In this sense, some research studies in Asian populations showed differences in the association of SUA to MetS by sex, with odds of MetS higher in women than men [27, 28]. Moreover, most studies focus on hyperuricemic populations, while in this study, the population was predominantly normouricemic; this fact could explain our results. On the contrary, some studies found that stratification by age changes the association of SUA to MetS. For example, a study in the Taiwanese military service population showed that elevated SUA women ≥40 years old presented higher odds of MetS than women <40 years old [29]. Another study in an older Italian population found that women with high levels of SUA showed a 58% increased risk of MetS, while in men, no association of SUA to MetS was found [30]. Aging and changes in the endocrine system may explain the susceptibility of women in the development of MetS and high levels of SUA [29]. Besides, estrogens show an inverse relationship with SUA levels, while testosterone increases them. This fact was observed in both sexes [22].

Several studies demonstrate the role of obesity and overweight in the development of cardiometabolic diseases [3–5]. Our results show that individuals with MetS present alterations in anthropometric parameters, BMI, body fat, and central obesity. However, the impact of SUA on MetS components was to increase the frequency of hypertriglyceridemia and not obesity. It is consistent with a longitudinal study in China that found that the highest quartile of SUA had 45.6% of cumulative incidence to hypertriglyceridemia [31]. In this sense, Jing Wu et al. showed that uric acid‐lowering therapy effectively improved serum cholesterol and triglyceride levels up to 80 mg/dL [32]. This may be explained by early metabolic changes such as activation of mitochondrial NADPH oxidase and inhibition of AMPK and AKT2, which alter lipid metabolism, mainly triglycerides [9]. Despite obesity is an important factor for the development of MetS, it is likely that disorders in lipid metabolism induced by uric acid develop earlier.

SUA is strongly associated with dyslipidemia and other metabolic disorders, having IR as a common factor [33]. We found that the high tertile of SUA is associated with elevated TyG in both sexes. In the same way, a study performed in Korea showed that the TyG index was significantly higher in the hyperuricemia than in the nonhyperuricemia group (8.96 vs. 8.54, *p* < 0.001) [34]. Furthermore, a study in ST-elevation myocardial infarction (STEMI) patients demonstrated that the highest quartile of TyG had the incidence of major adverse cardiovascular and cerebral events (MACCEs) which was higher [35]. On the contrary, Elizalde-Barrera et al. did not find any correlation between uric acid levels and homeostatic model assessment of *β*-cell function (HOMA 1B) (*r* = 0.102, *p* = 0.343) nor with HOMA of insulin resistance (HOMA 1IR) (*r* = 0.158, *p* = 0.117); when stratified by sex, women had a significant correlation with HOMA 1IR (0.278, *p* = 0.01), but not with HOMA 1B (0.138, *p* = 0.257) [36].

MetS is a group of insulin-related disorders that increases the risk of multiple diseases such as DM, hypertension, cancer, nonalcoholic fatty liver, chronic kidney disease, brain disorders, and susceptibility to infections [3, 9]. For this reason, MetS risk factors' research is important to establish therapeutic objectives and primary prevention. Reducing the incidence and prevalence of MetS may help to reduce the risk of developing chronic diseases that demand a high cost for the health system [1]. TyG is an obtainable and cost-effective noninsulin-based IR index that is very useful in primary healthcare [6]. The economic development of Peru has allowed for the adoption of some lifestyles similar to those in high-income countries, such as increased sedentarism, consumption of high-calorie foods, and development of metabolic disorders [37].

Assessing lifestyles with valid instruments is important to establish which factors are relevant to decreased incidence of MetS in susceptible populations such as health personnel, who have an environment with higher levels of stress, depression, burnout syndrome, bad sleep quality, and metabolic disorders [16, 17]. On the contrary, women appear to be more susceptible to some metabolic disorders than men [28, 38]. More research is necessary to establish stronger risk factors for MetS in this population.

## 5. Strengths and Limitations

To the best of our knowledge, this is the first study that assessed the association between SUA and MetS conducted in health personnel from Peru. The current study provides evidence on the dose-response relationship between SUA and MetS. Other strengths of our study included the adjustment of several potential confounders. However, some limitations should be highlighted. First, it was not possible to assess causality among the variables due to the nature of the cross-sectional design of the study. Second, although several confounding factors were controlled in the present analysis, there were confounding factors that we did not consider, such as glomerular filtration rate, liver enzymes, and antihyperuricemic agents. Third, information collected from the Plan for the Prevention of Communicable and Noncommunicable Diseases of Hospital de Huaycán was used, which could present some errors when filled in; however, a rigorous evaluation of the quality of the data was carried out to reduce the possibility of biased information. Finally, given that the study was conducted in a single hospital, there is potential for selection bias. Therefore, the results should not be generalized to the population.

## 6. Conclusions

We found evidence that SUA is positively associated with the prevalence of MetS in a population of health personnel at a public hospital from Peru, being the association strongest in women. Additionally, the increased concentration of SUA is an independent factor for hypertriglyceridemia and elevated insulin resistance markers with differences by sex. Longitudinal studies are needed to confirm these results and to determine significant risk factors for MetS considering the cultural environment of each population. More research in lifestyles with valid instruments and additional biomarkers' control could decrease the prevalence of MetS and other chronic noncommunicable diseases.

## Figures and Tables

**Table 1:** Prevalence ratio for MetS according to SUA tertiles by overall population and sex.

	Serum uric acid (tertiles)			Serum uric acid (mg/dL)
	T1	T2	T3	
	PR	PR (95% CI)	PR (95% CI)	PR (95% CI)
*Men*				
Metabolic syndrome				
Model 1^a^	1	2.19 (1.01–4.72)^*∗*^	2.11 (0.98–4.57)	1.40 (0.98–2.00)
Model 2^b^	1	2.36 (1.12–4.95)^*∗*^	1.73 (0.80–3.75)	1.29 (0.91–1.83)
Model 3^c^	1	1.79 (0.98–3.23)	1.31 (0.71–2.42)	1.23 (0.93–1.64)

*Women*				
Metabolic syndrome				
Model 1^a^	1	1.62 (0.94–2.80)	2.72 (1.69–4.38)^*∗∗*^	1.49 (1.26–1.76)^*∗∗*^
Model 2^b^	1	1.46 (0.85–2.50)	2.43 (1.50–3.93)^*∗∗*^	1.45 (1.23–1.72)^*∗∗*^
Model 3^c^	1	1.16 (0.68–1.96)	1.71 (1.07–2.74)^*∗*^	1.24 (1.01–1.52)^*∗*^

T1: low tertile; T2: middle tertile; T3: high tertile; CI: confidence interval; PR: prevalence ratio; ^*∗*^*p* < 0.05; ^*∗∗*^*p* < 0.01; ^a^nonadjusted; ^b^adjusted for age; ^c^adjusted for age, BMI, diabetes family history, physical activity, feeding habits, alcohol consumption, and smoking. Prevalence ratios and confidence intervals were calculated with Poisson regression with robust variance.

**Table 2:** Association between SUA tertiles and MetS components and insulin resistance.

Metabolic syndrome components	Men			Women		
	T1	T2, PR (95% CI)^a^	T3, PR (95% CI)^a^	T1	T2, PR (95% CI)^a^	T3, PR (95% CI)^a^
Central obesity	1	1.34 (0.64–2.82)	1.13 (0.52–2.43)	1	1.11 (0.64–2.82)	1.23 (0.79–1.94)
High blood pressure	1	0.94 (0.27–3.24)	1.00 (0.33–3.05)	1	0.67 (0.17–2.63)	1.53 (0.50–4.71)
Hypertriglyceridemia	1	2.27 (1.22–4.25)^*∗*^	1.94 (1.01–3.73)^*∗*^	1	1.32 (0.78–2.12)	2.02 (1.13–3.20)^*∗*^
Hyperglycemia	1	1.18 (0.66–2.10)	1.29 (0.73–2.25)	1	1.02 (0.68–1.54)	1.02 (0.67–1.53)
Low HDL-C	1	0.36 (0.09–1.46)	0.45 (0.13–1.60)	1	1.18 (0.69–2.01)	1.27 (0.75–2.14)
Elevated TG/HDL-C	1	1.82 (1.09–3.04)^*∗*^	1.67 (0.88–3.94)	1	1.24 (0.70–2.22)	1.44 (0.83–2.50)
Elevated TyG index	1	1.88 (1.08–3.30)^*∗*^	1.39 (0.75–3.30)	1	1.30 (0.85–1.99)	1.90 (1.30–2.76)^*∗∗*^

PR: prevalence ratio; 95% CI: 95% confidence interval; TG/HDL-C: triglyceride-to-HDL cholesterol ratio; TyG: triglyceride-glucose index. ^a^Adjusted for HbA1c, LDL-C, and alcohol consumption; ^*∗*^*p* < 0.05; ^*∗∗*^*p* < 0.01.

## Data Availability

The datasets used and analyzed for this study are available from the corresponding author upon reasonable request.

## Supplementary Materials

Supplementary Materials Table S1: title of data: data excluded by implausible or missing values. Description of data: data excluded from the Plan for the Prevention and Surveillance of Communicable and Noncommunicable Diseases by implausible or missing values. Table S2: title of data: general characteristics of the healthcare personnel. Description of data: general characteristics of the healthcare personnel by sex and MetS.
