# Supplementary material for: Serum Uric Acid Is Associated with Metabolic Syndrome and Insulin Resistance among Health Personnel from Peru
Source: J Nutr Metab. 2021 Nov 15;2021:9933319. doi: 10.1155/2021/9933319 (PMC8608523; doi:10.1155/2021/9933319)
Supplement: Supplementary Materials — Table S1: title of data: data excluded by implausible or missing values. Description of data: data excluded from the Plan for the Prevention and Surveillance of Communicable and Noncommunicable Diseases by implausible or missing values. Table S2: title of data: general characteristics of the healthcare personnel. Description of data: general characteristics of the healthcare personnel by sex and MetS. [file 9933319.f1.pdf]

Supplementary appendix

Table S1: Data excluded by implausible or missing values.

Variables	Overall population (n = 367)		
	**Missing**	**Implausible**	**Total excluded**
Glucose	18	1	19
Total cholesterol	0	2	2
HDL-C	5	11	16
LDL cholesterol	1	1	2
VLDL cholesterol	0	2	2
HbA1c	2	3	5
Serum Uric Acid	1	1	2
Waist circunference	11	0	11
Body fat percentage	2	1	3
Findrisc_1	1	1	2
Findrisc_2	1	0	1
Findrisc_6	0	1	1
Findrisc_8	0	3	3
Smoking	3	1	4
Age	1	1	2
Summatory	46	29	75
Percentage (%)	12.5	7.9	20.4

LDL-C, low density lipoprotein cholesterol; VLDL-C, very low-density lipoprotein cholesterol; HDL-C, high density lipoprotein cholesterol; HbA1c, glycosylated hemoglobin

Description of data: Data excluded from the Plan for the Prevention and Surveillance of Communicable and Non-Communicable Diseases by implausible or missing values.

Table S2: General characteristics of the healthcare personnel by sex and MetS

Variable	Overall population	Men (n=90)			Women (n=202)		
		No-MetS	MetS	P-value	No-MetS	MetS	p value
	(n=292)	(n=57)	(n=33)		(n=124)	(n=78)	
Age (years)	46.2 ± 10.6	45.6 ± 11.2	51.5 ± 10.2	0.014*	43.2 ± 10.1	49.0 ± 9.7	< 0.001**
Weight (Kg)	67.9 ± 13.0	70.9 ± 12.1	82.7 ± 12.8	< 0.001**	60.7 ± 9.8	71.2 ± 10.4	< 0.001**
BMI (Kg/m^2)	28.1 ± 4.3	26.8 ± 3.7	30.4 ± 3.7	< 0.001**	26.3 ± 3.7	30.6 ± 4.0	< 0.001**
Waist circumference (cm)	91.8 ± 10.8	91.7 ± 12.3	103.0 ± 7.4	< 0.001**	86.1 ± 9.2	96.3 ± 6.7	< 0.001**
Body fat (%)	33.0 ± 6.7	25.7 ± 5.6	29.4 ± 3.0	< 0.001**	33.8 ± 5.4	38.5 ± 4.4	< 0.001**
Glucose (mg/dL)	95.9 ± 32.7	93.6 ± 14.0	114.0 ± 48.9	0.027*	90.8 ± 32.8	98.2 ± 31.5	0.111
Cholesterol (mg/dL)	193.1 ± 37.0	187.0 ± 35.5	216.0 ± 34.7	< 0.001**	187.0 ± 33.4	199.0 ± 40.4	0.034*
Triglycerides (mg/dL)	153.7 ± 79.3	142.0 ± 77.2	234.0 ± 92.7	< 0.001**	116.0 ± 54.3	188.0 ± 69.4	< 0.001**
LDL-C (mg/dL)	114.3 ± 31.0	113.0 ± 28.7	129.0 ± 32.7	0.019*	111.0 ± 25.6	114.0 ± 37.8	0.460
VLDL-C (mg/dL)	29.07 ± 12.79	27.1 ± 11.5	40.5 ± 13.9	< 0.001**	24.5 ± 10.4	36.6 ± 12.8	< 0.001**
HDL-C (mg/dL)	49.8 ± 10.3	47.1 ± 7.7	47.5 ± 9.6	0.818	53.0 ± 11.2	47.8 ± 9.5	< 0.001**
HbA1c (%)	6.0 ± 1.0	5.8 ± 0.6	6.6 ± 1.8	0.028*	5.8 ± 0.8	6.2 ± 1.0	< 0.001**
Uric acid (mg/dL)	3.9 ± 1.0	4.6 ± 0.8	4.9 ± 0.9	0.054	3.3 ± 0.8	3.8 ± 0.8	< 0.001**
SBP (mmHg)	108.1 ± 13.4	110.0 ± 12.0	123.0 ± 11.5	< 0.001**	103.0 ± 11.1	109.0 ± 13.4	< 0.001**
DBP (mmHg)	68.8 ± 10.5	69.3 ± 9.8	80.3 ± 10.7	< 0.001**	65.0 ± 8.8	69.7 ± 9.8	< 0.001**
Uric acid tertiles (%)							
T1[a]	100 (34.2)	26 (78.8)	7 (21.2)	0.068	57 (78.1)	16 (21.9)	< 0.001**
T2[a]	96 (32.9)	15 (53.6)	13 (46.4)		40 (64.5)	22 (35.5)	
T3[a]	96 (32.9)	16 (55.2)	13 (44.8)		27 (40.3)	40 (59.7)	

Data expressed as mean ± standard deviation or number (percentage). MetS, Metabolic Syndrome; BMI, Body mass index; LDL-C, Low density lipoprotein cholesterol; VLDL-C, Very lowdensity lipoprotein cholesterol; HDL-C, High density lipoprotein cholesterol; HbA1c, Glycosylated hemoglobin; SBP, Systolic blood pressure; DBP, Diastolic blood pressure; T1, Low tertile; T2, Middle tertile; T3, high tertile. *p <0.05, **p<0.01, [a]Interval of uric acid depend of the group (overall population, male or women).

The p value was calculated for the numeric variables with the Welch Two Sample t-test, and for the categorical variables with Chi-Square test.
